# Editorial Note

**Published:** 1912-03

**Authors:** 


### Editorial Note.

**Dr. J. B. Siddall.**

We learn that the Emperor of Japan has performed a most graceful act in conferring the Order of the Rising Sun on Dr. Joseph Bower Siddall, M.D., C.M. Aberd., D.P.H. Camb., formerly of St. Thomas's Hospital, and now residing at Malvern.

Dr. Siddall was formerly House Physician and afterwards Physician to the Bristol General Hospital. We congratulate our old Bristol *confrère* on this recognition (although so long delayed by red-tape difficulties) of his services to Japan in his early years in 1868 and 1869, when he established a large hospital in Tokio, which became the nucleus of a medical school at which many who are now leaders in the medical work of Japan were some of the earliest students. He rendered valuable service in aiding the Japanese wounded in their revolutionary war at a time when wounded prisoners were usually put to death. The "Rising Sun" holds the same place in Japan as the Order of the Bath does with us, and is the highest honour that can be conferred on subjects not of noble birth for public services, and Dr. Siddall is the only member of the profession in England who holds this decoration.

